# Teams in Transition: A Three-Wave Longitudinal Study of Reflection, Implicit and Explicit Coordination and Performance Improvements

**DOI:** 10.3389/fpsyg.2021.677896

**Published:** 2021-06-07

**Authors:** Udo Konradt, Michaéla C. Schippers, Sabrina Krys, Ashley Fulmer

**Affiliations:** ^1^Work and Organizational Psychology, Institute of Psychology, Kiel University, Kiel, Germany; ^2^Department of Technology and Operations Management, Rotterdam School of Management, Erasmus University of Rotterdam, Rotterdam, Netherlands; ^3^J. Mack Robinson College of Business, Georgia State University, Atlanta, GA, United States

**Keywords:** team reflection, implicit coordination, explicit coordination, performance improvement, latent growth modelling

## Abstract

Research has shown that team reflection is a critical transition process for coordination processes and team performance, but our understanding of its dynamics and relationship to action processes and performance is incomplete. The goal of the present study was to examine the long-term change in reflection in teams over time and explore whether these changes are related to implicit and explicit coordination processes and performance improvement. Drawing on the recurring phase model of team processes and team reflexivity theory, we hypothesized that team reflection is at least stable or increases over time for dissimilar tasks, that reflection trajectories are positively associated with implicit and negatively associated with explicit coordination in the later phases, and that implicit coordination mediates the relationship between team reflection and performance improvement. This model was tested in a three-wave longitudinal study (*N* = 175 teams) over a 2-months period. Results from growth curve modeling and structural equation modeling provided support for our hypotheses.

## Introduction

Even though team processes and actions are inherently dynamic phenomena (Ancona et al., 2001; Cronin et al., 2011; Kozlowski, 2015), the research on teamwork has often employed a rather static approach toward team functioning. Therefore, in their review of a century of teamwork research, Mathieu et al. (2017) concluded that “there is a path dependence to teamwork that implies we really cannot fully appreciate or understand the critical variances that are involved unless we take time–in its various incarnations–into account” (p. 462). Accordingly, we address this issue by examining the dynamics of transition processes (i.e., team reflection) and its relationship to action processes (i.e., implicit and explicit team coordination) and outcomes (i.e., team performance improvement).

Tjosvold (1991) noted that “experience itself does not teach; people learn from reflecting on their experience” (p. 189). Much of team learning occurs via reflection (Schippers et al., 2013; Gabelica et al., 2014), which refers to the extent to which group members consciously reflect upon and communicate about the group’s objectives, strategies (e.g., decision-making), and processes (West, 2000). Team reflection (also known as after-event-reviews or team debriefs) has shown to be critical for team outcomes, including effectiveness (Widmer et al., 2009; Schmutz et al., 2018), performance (Schippers et al., 2008; Villado and Arthur, 2013; Konradt et al., 2015), and innovation (Tjosvold et al., 2004; Schippers et al., 2015). In addition, research has demonstrated that team reflection reduces work-based strain through enhanced control and support (Chen et al., 2018).

**FIGURE 1:**
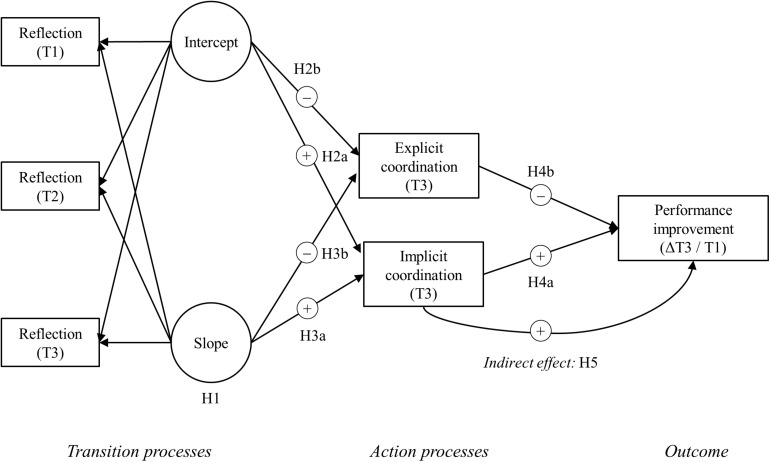
Theoretical model of the relationships between change in transition processes, subsequent action processes, and team performance improvements.

While evidence suggest benefits of reflection for team performance-related outcomes (for reviews, see Konradt et al., 2016; Schippers et al., 2018; Otte et al., 2019), surprisingly little is known about how team reflection develops over time, particularly over longer periods of time (Bush et al., 2018). Studies that rely on cross-sectional (“snapshots”) or short-term pre-post designs are not able to capture this change, only show parts of the whole and may result in incorrect conclusions (Kolbe and Boos, 2019; Kozlowski, 2015). This is all the more surprising because widely used team development and episodic teamwork theories (Gersick, 1988; McGrath, 1991; Marks et al., 2001) conceptualize teamwork as a phenomenon that changes over time. Marks et al. conceive team reflection as a transition process that serves as a precursor of subsequent action processes and outcomes. However, the relationship between reflection as a transition process and subsequent action processes that mediate the reflection–outcome relationship, such as team coordination (Rico et al., 2008), have rarely been examined. Thus, the main contribution of this research is to take an initial step in identifying how reflection in teams develops over time in the long-term, and how it relates to subsequent team coordination processes and performance. Our research model is depicted in Figure 1.

## Dynamics in Team Reflection: A Transition Process

In their recurring phase model of team processes, Marks et al. (2001) distinguished between team processes such as reflection or coordination and emergent states such as shared mental models. Team processes help members to utilize various resources to achieve their collective goals, and these have further been delineated into transition processes, by which team members direct, align, and evaluate what they are doing (e.g., reflection) to accomplish the team’s task, and action processes, which translate the results of the transition processes into action (e.g., coordination). The proximal outcome(s) of an action phase serve as future inputs for a next transition phase which reflects the principle of a causal feedback loop (Ilgen et al., 2005; Konradt et al., 2016). For this reason, the baseline level of a particular process and the rate at which the process develops over time are important predictors of team behavior and outcomes. Team reflection is a key process that strongly influences team learning and performance. It takes place during transition phases (Schippers et al., 2013; Gabelica et al., 2014) and can be defined as the extent to which team members reflect upon and discuss the team’s objectives, strategies (e.g., decision-making) or processes (West, 2000).

Even though the recurring phase model of team processes (Marks et al., 2001) was published 20 years ago, reflection research has rarely examined one of the core ideas of this model: that teams go through different episodes, which creates trajectories of team processes (i.e., different baseline level and rate of change). Recent research has explored the implications of time on team processes. In two longitudinal studies using business simulation tasks, Konradt and Eckardt (2016) and Li et al. (2020) showed that team reflection generally decreased over time when tasks remained similar over time. They argued that when tasks do not vary much, the team’s previous experience of performing similar tasks is more relevant for and applicable to the present task, enabling team members to draw on existing routines. In this situation, tasks can become relatively automatic and effortless (see also Ackerman, 1987) and “team members may see limited need to discuss, debate, and reflect about needed actions” (Schmutz et al., 2018, p. 5). However, in today’s dynamic environment, tasks are rarely constant or homogeneous, and heterogeneous tasks add uncertainty and ambiguity (cf. Schmutz et al., 2018). In their integrative review of transition processes in teams, Bush et al. (2018) argue that the level of similarity of the tasks has implications to the transition activities and team effectiveness. Specifically, they proposed that the more heterogeneous the tasks, the more relevant are transition activities to effectiveness in the subsequent task in terms of changes to the team’s mindset or approach. Also, heterogeneous tasks require different sets of activities and strategies (Harrison et al., 2003), which makes teams hold the level of reflection high or even increase it. Consequently, Schmutz et al. (2018) demonstrated that in-action team reflection that occurs also during performance events tends to increase as action progresses. Hence:

**Hypothesis 1:** Teams working on heterogeneous tasks (i.e., low levels of similarity) show stable or increasing levels of reflection over time.

## Team Coordination as an Action Process

Models of transition processes in teams (West, 2000; Marks et al., 2001) suggest that reflection is predictive of the subsequent action processes of adaptation and implementation, which should in turn predict and explain team performance. During action phases, team members work to complete the task at hand, implement strategies, and monitor progress to reach their common goals (Marks et al., 2001). Despite the theoretical and practical relevance of existing studies on team reflection, it has been pointed out that almost all have focused on the relationship between reflection and performance, leaving out the specific action processes involved (and thus also the mediating processes) that would explain how team reflection is related to team performance (Konradt et al., 2015).

One such action process that involves adaptation and implementation is coordination, defined as “the process of orchestrating sequence and timing of interdependent actions” (Marks et al., 2001, p. 367). Rico et al. (2008) remarked that a distinction should be made between different coordination processes according to how much teams relied on direct communication exchange between team members. They differentiated between *explicit coordination*, namely the explicit request and exchange of information to coordinate the activities of the individual team members, and *implicit coordination* (see also tacit coordination in Wittenbaum et al., 1998). Drawing on team cognition research, they contended that implicit coordination occurs “when team members anticipate the actions and needs of their colleagues and task demands and dynamically adjust their own behavior accordingly, without having to communicate directly with each other” (p. 164). If such procedures are covered by a shared team mental model (Mohammed and Dumville, 2001) and collective memory system (i.e., transactive memory system; TMS; Lewis, 2004; Lewis et al., 2005), implicit coordination will enable team members to make reliable forecasts of how others will react to plans and particular levels of performance and to anticipate what other team members are likely to do or need, without any explicit discussion of who should do what (Kolbe et al., 2013). Shared mental models are defined as “an organized understanding or mental representation of knowledge that is shared by team members” (Mathieu et al., 2005, p. 38) and TMS refer to “a collective memory system for encoding, storing, retrieving, and communicating group knowledge” (Lewis et al., 2005, p. 581; Wegner, 1986; Hollingshead, 2001). Both constructs have been shown to be an important precursor of implicit coordination (Rico et al., 2008; Mohammed et al., 2010) and team coordination quality (Moreland and Myaskovsky, 2000; Wiedow et al., 2013; Ellwart et al., 2014). Evidence also suggests that shared mental models reduce team reflection (van Ginkel et al., 2009), and that team reflection has a positive effect on team mental model similarity (Tesler et al., 2018). In addition, Oertel and Antoni (2015) demonstrated a positive relationship between reflection and transactive memory development.

Implicit coordination requires shared cognitions and a collective memory, which are developed through team reflection, that enable each team member to act in accordance with the needs of the other team members (Rico et al., 2008). Teams that have not established a shared cognition and collective memory have to coordinate explicitly by constantly communicating their actions. In fact, a field study with medical teams demonstrated that the amount of explicit coordination decreased, while implicit coordination increased (Riethmüller et al., 2012). The authors argued that implicit coordination enables teams to “enhance team performance by freeing up cognitive resources that can instead be used for patient care, revealing adaptive coordination as a skill developed through repeated group interaction” (p. 16). Consequently, we assume that teams who discuss and evaluate their work on the task (e.g., who reflect on their coordination processes) should be able to make more extensive use of implicit coordination across time. In contrast, teams that do not discuss the way they coordinate are not able to anticipate each other’s actions, cannot be proactive, and will therefore have to use more explicit coordination. This leads us to the following hypotheses:

**Hypothesis 2:** Teams with higher baseline levels of reflection will show higher levels of implicit coordination (a) and lower levels of explicit coordination (b) at later points in time.

**Hypothesis 3:** Teams that show a greater increase in reflection will show higher levels of implicit coordination (a) and lower levels of explicit coordination (b) at later points in time.

## Team Performance Improvement

As discussed above, coordination has been conceived as a central process for effective team functioning (Stewart, 2006; LePine et al., 2008). More specifically, previous research posited that implicit coordination, especially in the later phases of teamwork in response to changes in the team’s task environment, is more beneficial to team performance than explicit coordination (Marques-Quinteiro et al., 2013). Explicit coordination takes time and requires cognitive resources, whereas implicit coordination “does not depend on verbal communications and may thus conserve cognitive resources for attending to immediate decisions and actions” (Marques-Quinteiro et al., 2013, p. 196). This implies that implicit coordination has an advantage over explicit coordination, especially in terms of efficiency (as opposed to effectiveness); it means that the team has more time and cognitive resources at its disposal, which ultimately leads to better team performance. This assumption has also been substantiated empirically (Butchibabu et al., 2016). For example, evidence from a computer-based shared interface experiment suggests that teams, which foster the shared cognition, experienced more implicit coordination, and showed higher performance than the non-shared interface control group (Lowry et al., 2013). Thus, it is assumed that teams that coordinate their actions implicitly perform better than teams that have to coordinate explicitly (Marques-Quinteiro et al., 2013).

Therefore, we expect there to be a positive relationship between implicit coordination and team performance. In contrast, teams that use explicit coordination are likely to be less efficient and therefore less effective overall, as they have to spend more time on coordinating their actions and have less time for task-related activities such as problem solving.

When teams are conceptualized as dynamic systems, performance outcomes can be measured at multiple points in time. We argue that measuring changes in performance at intervals throughout a process provides a more valid measure of learning and overall team success than looking solely at the final outcome, because it reflects the team’s progress over time (Konradt et al., 2015; Otte et al., 2018). Also, by recording the change in performance for each team, this measure of performance improvement also eliminates alternative explanations that might focus on the teams’ disparate starting levels of performance. Accordingly, we hypothesize the following:

**Hypothesis 4:** (a) Implicit coordination at later points in time will be positively related to improvements in team performance, whereas (b) explicit coordination at later points in time will be negatively related to performance improvements.

The recurring phase model (Marks et al., 2001) posits that a transition process is connected to an outcome via an action process. However, although the link between reflection (transition process) and performance (outcome) is by now quite well established (Schippers et al., 2018), previous empirical research has rarely provided evidence of the action processes that mediate the effect of reflection on team performance (improvement). We thus expect a mediation effect of implicit coordination between team reflection and performance improvement. The mediation effect of implicit coordination is likely for teams that have developed a shared understanding of their tasks (i.e., shared mental models) and knowledge sharing and retrieval processes among team members (i.e., TMS). Evidence suggests that the effects of team reflection on team performance improvement are mediated by a path from shared team mental models to shared task mental models and to adaptation (Konradt et al., 2015).

Because shared mental models and TMS are important precursors of implicit coordination (Rico et al., 2008; Mohammed et al., 2010) and team coordination quality (Moreland and Myaskovsky, 2000; Wiedow et al., 2013; Ellwart et al., 2014), and because shared mental models, TMS, as well as implicit coordination are associated with higher performance (Liang et al., 1995; Lowry et al., 2013; Marques-Quinteiro et al., 2013; Konradt et al., 2015), we expect that implicit coordination will mediate the relationship between the parameters of team reflection trajectories (i.e., baseline level, that is the intercept in team reflection, and growth, that is the rate of change or slope in team reflection) and team performance improvement. Thus, we hypothesize:

**Hypothesis 5:** Implicit team coordination at later points in time will mediate the effect of the baseline level and rate of change of team reflection on performance improvement.

## Materials and Methods

### Data and Sample

Participants in this study were first-year business students at a Dutch university. Students were enrolled in a 2-months research methods class, at the beginning of which they formed self-selected teams to complete different kinds of team tasks. The data-gathering process was similar to the process reported by Schippers (2014), although was undertaken with a different cohort. Teams were examined at three points in time. The students completed online surveys about their team, measuring team reflection after the first task (T1), in the middle of the class (T2), and after the final task (T3), team performance at the beginning (T1) and after the final task (T3), and implicit and explicit coordination at T3.

We focused on the first and the last task, because these represent the end points of the time continuum and best reflect the improvement over time. The first task required to read a case and conduct a literature review of one of the subjects mentioned in it. Teams had to use various internet databases, such as Google Scholar and JSTOR. The teams were expected to judge the quality of the different sources and articles they found, and the most important part of this task was to compare the findings of the articles.

In the last task, students were asked to read a business case and formulate relevant research questions. They were then asked to choose a particular research method to answer the research question (e.g., experiment, case study, survey study). They had to give convincing scientific arguments for their choice. In addition, they had to indicate what kind of answers could, or could not, be expected, based on the chosen research method (e.g., theory building vs. theory testing). They also had to indicate what type of research strategy would be least suitable for answering the research question, and to explain why. Finally, they were asked to make a recommendation based on the case.

In total, the teams worked together for about 2 months. They worked in three- or four-person teams; in the final sample, 85 teams were three-person teams, and 90 teams were four-person teams. A total of 196 teams completed the study. Twenty-one teams, in which only one or two members provided data, were omitted from the analyses to estimate unbiased standard errors. The final sample thus consisted of 175 teams (*n* = 615 team members). One-way ANOVAs revealed no significant differences among the initial and the final sample in any of the team variables measured (see below).

### Procedure

Data was collected using online survey sent out by two research assistants. Neither the author nor the assistants were involved in teaching the class in which the team tasks were undertaken. Students were contacted by email and asked to fill out an online questionnaire but were also given the opportunity to opt out. The teams received the grades for their tasks after the questionnaires had been filled out ensuring that the feedback does not interfere with responding. Participants were briefed afterward about the purpose of the research. The teachers were not aware of the purpose of the research; they were only told that a study on teamwork would take place during the course.

### Measures

#### Team Reflection

Team reflection within teams was measured using the four-item measure from Schippers et al. (2007) discussing processes subscale of their reflection measure that is based on the items from Swift and West (1998). This measure is similar to the team reflexivity measure used in Yang et al. (2020) and De Jong and Elfring (2010), which is based on the items from Carter and West (1998). In contrast to Yang et al. and De Jong and Elfring, the scale of Schippers et al. omits the item on adaptation because adaption is not a part of team reflection. Example items are “We regularly discuss whether the team is working effectively” or “The team often reviews whether it’s getting the job done” (1 = *totally disagree*; 5 = *totally agree*). Cronbach’s alphas from T1 to T3 were 0.75, 0.76, and 0.76, respectively.

#### Explicit Coordination

This was measured using the five-item coordination subscale developed by Lewis (2003). Example items are “Our team needed to backtrack and start over a lot” or “There was much confusion about how we would accomplish the task” (1 = *totally disagree*; 5 = *totally agree*). In contrast to the original scale, high values imply more explicit coordination. Therefore, all items are coded in the opposite direction of the original version. Cronbach’s alpha at T3 was 0.87.

#### Implicit Coordination

Implicit coordination was measured using a 17-item scale based on the two basic components in implicit coordination proposed by Rico et al. (2008). This measure assesses the extent to which team members continuously adopt to mutually adapt their behavior and anticipate each other’s tasks, actions, and needs. Sample items are “When I am under time pressure, other team members proactively help me.” or “I often consider other team members’ workload to see if they need some assistance” (1 = *totally disagree*; 5 = *totally agree*; Cronbach’s alpha at T3 was 0.84). The scale items appear in the Appendix.

#### Team Performance Improvement

Team performance improvement was measured using two standardized grade scores (for the first and last task) given by the instructors, who were not associated with the data collection. A score for each task was awarded to each team and was assigned to all team members for grading purposes. This procedure ensured a condition of common fate, or high outcome-interdependence among the team members. This would ensure they focused on team goals as opposed to individual goals (DeShon et al., 2004). Team performance improvement was represented as a latent variable of the standardized scores for the last task, controlling for the standardized score for the first task as a covariate. The grading scale ranged from 1 to 10, with 1 denoting “bad” and 10 denoting “excellent.” Students with a mean overall grade lower than 5.5 failed the course. The average score for the first task was 7.43 (*Min* = 4.00, *Max* = 9.70, *SD* = 1.06), and the average score for the last task was 6.93 (*Min* = 4.00, *Max* = 9.00, *SD* = 1.08).

### Data Analyses

#### Discriminant Validity

Following the recommendations of Podsakoff et al. (2003), we collected data at different times for several weeks. To examine whether common-method variance might be influencing our results, we considered an additional structural model which included a single unmeasured latent factor. The results indicated only minor changes in our hypothesized model, with the relationships remaining consistent with our hypotheses. Finally, a confirmatory factor analysis was run to examine the discriminant validity of the three constructs at T3 (i.e., reflection, implicit coordination, and explicit coordination). Our hypothesized three-factor model (reflection, and explicit and implicit coordination) was considered an acceptable fit to the data (cf. Bentler and Hu, 1995) (χ^2^ = 244.33, *df* = 150, *ns*., comparative fit index (CFI) = 0.94, Tucker-Lewis index (TLI) = 0.92, root-mean-square effort of approximation (RMSEA) = 0.05). It fitted significantly better than alternative models, including one in which only a single general factor was specified (χ^2^ = 591.04, *df* = 153, *ns*., CFI = 0.73, TLI = 0.66, RMSEA = 0.12). From these results we concluded that the measures captured related but distinct constructs.

#### Interrater Agreement

To justify aggregating individual-level data to the team level, we calculated *r*^∗^_*wg(J)*_ (Lindell et al., 1999) and *a*_*WG(J)*_ values (Brown and Hauenstein, 2005) using the multilevel R package provided by Bliese (2016). *a*_*WG(J)*_ values ranged from 0.69 to 0.74, and *r*^∗^_*wg(J)*_ ranged from 0.61 to 0.71. These results indicated acceptable agreement within groups to aggregate data at the team level (cf. Brown and Hauenstein, 2005; LeBreton and Senter, 2008; Cohen et al., 2009). Additionally, we calculated the ICC(1) and ICC(2) values for each measure (see Table 1). According to Woehr et al. (2015), it is preferable to evaluate agreement in comparison to levels typically found in the literature for similar constructs. All three ICC(1) values for team reflection lie between 0.00 and 0.10, which is consistent with 22.4% of previous research on group constructs. The ICC(1) for implicit coordination lies in the third category (0.11–0.20), which comprises 28.97% of previous research. The ICC(1) for explicit coordination aligns with 16.21% of previous research and lies in the fifth category (0.31–0.40), so we consider our ICC(1) values to be acceptable. Regarding the ICC(2) values, we observe that the agreement values for team reflection are relatively low. Only 1.26% of the previous research is in the range of 0.11 and 0.20. The ICC(2) for implicit coordination is also in a category (0.31–0.40) that includes only 6.28% of the previous research. For explicit coordination, the situation is different: here, the ICC(2) lies in a category (0.61–0.70) that encompasses 15.06% of the previous research. Bliese (1998) argued that low ICC(2) values attenuate observed relationships and therefore limit the ability to identify relationships between variables at the group level. Also, the unreliability at group level reinforces Type II errors (Hofmann and Jones, 2005), which leads to more conservative analyses. Snijders and Bosker (1999) noted that the ICC(2) values tend to underestimate true reliability in small teams. This should work against us in terms of supporting our hypotheses. Given all the evidence on the ICC(1), ICC(2), *r*^∗^_*wg(J)*_, and *a*_*WG(J)*_, we proceeded to create aggregate measures of team reflection, implicit and explicit coordination, noting that the reliability of these means might be limited. Therefore, the results presented using these measures should be interpreted as conservative considering the possible attenuation.

**TABLE 1:** Interrater agreement for aggregated team variables, descriptive statistics, correlations, and reliability coefficients.

**Variable**		r*_wg_(J)	***a_WG_(J)***	**ICC(1)**	**ICC(2)**	***M* (*SD*)**	**1**	**2**	**3**	**4**	**5**	**6**
1	Team Reflection at T1	0.61	0.69	0.04	0.14	2.65 (0.39)	(0.75)					
2	Team Reflection at T2	0.61	0.69	0.07	0.20	2.81 (0.37)	0.47**	(0.76)				
3	Team Reflection at T3	0.63	0.71	0.06	0.18	2.82 (0.40)	0.43**	0.63**	(0.76)			
4	Explicit coordination at T3	0.70	0.74	0.36	0.65	3.67 (0.46)	–0.08	−0.28**	−0.28**	(0.87)		
5	Implicit coordination at T3	0.71	0.74	0.15	0.36	3.67 (0.26)	0.14	0.13	0.17*	0.44**	(0.84)	
6	Performance improvement^a^	−	−	−	−	0.49 (1.48)	–0.01	0.11	0.01	0.01	0.06	−

*N = 175 teams. Numbers on the diagonal are reliability estimates (α) where appropriate.*

^*a*^Because performance improvement was modeled as a latent variable, correlations for performance improvement were calculated in a hierarchical regression analysis, in which each variable was regressed on the residual of the last grade after controlling for the first grade.

*p < 0.05; **p < 0.01 (two-tailed).

#### Framework for Hypotheses Testing

To analyze the data, we used latent growth curve modeling (LGC; Bollen and Curran, 2006; Curran et al., 2010) with Mplus 8.4 (Muthén and Muthén, 1998–2020). LGC modeling is used to estimate the patterns of change, also called time trends, time paths, growth curves or latent trajectories. More specifically, the intercept (i.e., starting point or baseline) and the slope (i.e., rate of change or growth) are estimated as latent factors. We used a Bayesian estimator because of its fundamental advantages over the traditional frequentist approach in statistical modeling and data analysis for the handling of non-normal, skewed posterior distributions, and more complex models (Muthén and Asparouhov, 2012; Zyphur and Oswald, 2015). The Bayesian estimator was used with two Markov chain Monte Carlo chains and 50,000 iterations. We assigned uninformative prior distributions to model parameters (see standard Mplus settings). To evaluate the convergence behavior of the Markov chains, we inspected the trace plots and the autocorrelation functions of all estimated parameters.

A well-specified model fit was indicated by the posterior predictive p-value (PPP), which indicated a good fit when it is equal to or higher than 0.05, and by a posterior predictive checking (PPC) 95% credibility interval (CI) for all estimated effects in the structural equation model, in which a negative lower bound is considered to be one indicator of good model fit. The deviance information criterion (DIC) was used to compare the models, with small DIC values indicating a better fit. The analyses were conducted on a significance level of α = 0.05 (one-tailed).

Before testing our hypotheses, we examined the change in the focal variable (i.e., team reflection) and compared two latent growth models. The two models were a (1) fixed linear growth model and a (2) free-form model (i.e., unstructured model; Grimm and Ram, 2012) with loadings from the slope at T1 fixed to 0, at T2 free, and at T3 fixed to 1. In contrast to the fixed model [PPC 95% CI (0.295, 29.910), PPP = 0.020, DIC = 349.774, RMSEA = 0.171, CFI = 0.891], the unstructured model fitted the data very well [PPC 95% CI (−10.546, 15.298), PPP = 0.390, DIC = 338.417, RMSEA = 0.028, CFI = 0.998]. We therefore decided to continue the analysis with the unstructured model.

## Results

Means, standard deviations, correlations, and reliabilities are presented in Table 1. The unstructured growth model for hypotheses testing provided an excellent fit to the data [PPC 95% CI (−21.72, 25.80), PPP = 0.40, DIC = 1495.86, RMSEA = 0.00, CFI = 1.00]^1^. Hypothesis 1 predicted that teams show stable or increasing levels of reflection over time. The means of the latent factors showed that the trajectory had a statistically significant intercept of 2.65 [*Posterior SD* (*PSD*) = 0.03, CI (2.59, 2.72), *p* < 0.001] and a significant positive slope of 0.16 units for each period [*PSD* = 0.03, CI (0.10, 0.23), *p* < 0.001]. Overall, the model-implied mean rate of reflection increased significantly, from 2.65 to 2.97, over the period of the study, thereby supporting Hypothesis 1. The model also provided evidence of significant variance components in both intercept [μi = 0.16, *PSD* = 0.02, CI (0.12, 0.21), *p* < 0.001] and slope factors [μs = 0.11, *PSD* = 0.03, CI (0.04, 0.17), *p* < 0.001], which indicated that there were significant individual differences in baseline levels and in rates of growth over time. The intercept (i.e., baseline) and the slope (i.e., growth) were statistically significantly and negatively related [β = −0.64, *PSD* = 0.11, CI (−0.76, −0.37), *p* < 0.01].

Hypothesis 2a, which predicted that teams with high baseline levels of reflection would show higher levels of implicit coordination at later points in time, was supported [β = 0.26, *PSD* = 0.12, CI (0.02, 0.49), *p* < 0.05]. In support of Hypothesis 2b, teams with higher baseline levels of reflection showed lower levels of explicit coordination at later points in time [β = −0.39, *PSD* = 0.12, CI (−0.64, −0.15), *p* < 0.001]. Hypothesis 3b, which predicted that teams with a greater increase in reflection would show lower levels of explicit coordination at later points in time, was also supported [β = −0.49, *PSD* = 0.13, CI (−0.75, −0.230, *p* < 0.001]. However, Hypothesis 3a, which predicted that teams with a greater increase in reflection would show higher levels of implicit coordination at later points in time, was not supported [β = 0.16, *PSD* = 0.14, CI (−0.12, 0.43), *ns*.]^2^.

Hypothesis 4a, which predicted that implicit coordination at later points in time would be positively related to performance improvement, received support [β = 0.55, *PSD* = 0.24, CI (0.07, 1.01), *p* < 0.01], as did Hypothesis 4b, which predicted that explicit coordination at later points in time would be negatively related to performance improvement [β = −0.70, *PSD* = 0.22, CI (−1.07, −0.21), *p* < 0.01]^3^. Finally, Hypothesis 5, which predicted that implicit coordination at later points in time would mediate the relationship between baseline levels of reflection and performance improvement, was supported [β = 0.13, *PSD* = 0.09, CI (0.00, 0.36), *p* < 0.05], although implicit coordination at later points in time did not mediate the relationship between increases in reflection and performance improvement [β = 0.07, *PSD* = 0.09, CI (−0.07, 0.30), *ns*.].

## Discussion

The purpose of this study was to explore the long-term dynamic nature of reflection in teams and its relationship to team coordination and team performance improvement in a three-wave longitudinal study over a 2-months period. Drawing on dynamic conceptualizations of the recurring phase model of team processes and reflexivity theory, our study findings contribute to the field of team research in four different ways. First, we showed that teams working on heterogeneous tasks typically increase their use of reflection over time. Teams that started with lower baseline levels of reflection increased their reflection over time to a greater extent than teams that started with higher baseline levels. Second, we found that teams with a high baseline level of reflection exhibited more implicit coordination 2 months later, while they exhibited less explicit coordination at that point. Furthermore, we showed that teams that increased their level of reflection over time exhibited less explicit coordination 2 months later than teams that did not increase their level of reflection. However, we did not observe a relationship between the increase in team reflection over time and implicit coordination at the end of the teamwork. Third, we demonstrated that teams with high levels of implicit coordination after 2 months showed greater improvements in performance over time, but the level of improvement over that period was less in teams with high levels of explicit coordination. Finally, implicit coordination mediated the relationship between the baseline level of reflection and team performance improvement but not the relationship between increases in reflection and team performance improvement (due to there being a non-significant relationship between increases in reflection and implicit coordination). We discuss these findings in more detail, with a focus on the main theoretical contributions and practical implications.

### Theoretical Implications

Our research provides new insights into the role of reflection dynamics in team behavior and team performance improvement. First, it adds to prior studies (Konradt et al., 2016; Schippers et al., 2018) by showing that teams working on heterogeneous tasks increase their reflection over time. This finding complements previous research (Konradt and Eckardt, 2016; Li et al., 2020), who demonstrated that teams that undertake homogeneous tasks show an overall pattern of decreasing reflection over time. The authors argued that, for homogeneous tasks, positive performance feedback leads to a reduction in reflection and to the use of closed action strategies, which enhance knowledge integration rather than knowledge generation (Harrison et al., 2003; Bush et al., 2018). For heterogeneous tasks, however, positive performance feedback does not provide any indication of what the team may need to do next, and open action strategies that support knowledge generation are thus required (Bush et al., 2018). Together, these findings suggest that the type of task is key in determining the extent to which teams reflect and may account for reflection dynamics. We also showed that the variance in slope and intercept was significant and that the intercept and slope were negatively related, suggesting that there could be different subgroups in the population characterized by different trajectories. Inspection of the slopes of random teams showed that while some teams have a positive slope with lower intercepts, there are also other teams that have a negative slope with higher intercepts. There were also teams that showed no change in team reflection and had a lower intercept. This would lead to the question of whether different groups can be identified and whether group membership can be predicted by other variables. For example, some teams might be characterized by a relatively high level of reflection from the beginning to the end of teamwork, while other teams might be characterized by a relatively low level of team reflection at the beginning of teamwork and a steep increase in reflection over time. Konradt and Eckardt (2016) showed, for example, that, due to ceiling effects, teams that initially reflected a great deal were not likely to be able to increase their reflection over time to a great extent compared to teams that reflected less at the start. Person-centered approaches (see Howard and Hoffman, 2018) thus could complement variable-centered approaches in terms of identifying different subgroups and turning away from the “average” team to a more detailed analysis of different team reflection trajectories.

Second, our research highlights the fundamental dimension of time in team transition and action processes, which in turn affect team performance. Since the dependencies between transition processes, action processes and team performance take time to evolve, it is essential to focus on improvements over longer periods of time, rather than expecting short-term progress or an immediate return. Likewise, this study supports the general finding that the parameter of reflection trajectories (i.e., baseline level and growth) act as predictors of team action processes (i.e., explicit and implicit coordination), which in turn predict outcomes (i.e., performance improvements), although we did not find significant associations between the slope of reflection and implicit coordination at a later point in time. A possible reason for this could be that teams with a low baseline level of reflection (and therefore positive growth in reflection) were not able to increase their reflection sufficiently to allow them to coordinate implicitly and might have needed more time to reach that tipping point.

Consistent with previous theories (McGrath, 1991; Marks et al., 2001) and the sparse research (Riethmüller et al., 2012; Lowry et al., 2013), our findings also indicate the significance of implicit coordination for understanding how team reflection over time affects improvements in team performance. Specifically, our results show that managers should pay particular attention to ensuring that teams reflect in the early stages of collaboration so that they can benefit from time-saving implicit coordination and improve their performance. We highlight how explicit coordination is double-edged in terms of its effect on performance and performance improvement: Marques-Quinteiro et al. (2013) argued that it is essential for task processing and performance and can thus be seen as effective, but it is also costly in terms of time and money and may seem to hamper performance improvement, so might deemed to be inefficient. Consistent with previous work (Riethmüller et al., 2012), we expect that, in the long run, high performance teams reduce their explicit coordination and intensify their implicit coordination.

### Limitations and Directions for Future Research

Although this study contributes in important ways to the team literature, it has some limitations and there are several ways in which our findings could be expanded upon in future research. Two major directions for future research that seem particularly promising include broadening the measurement of processes and states in teams and expanding the focus on other patterns of change.

A first set of limitations of the study pertains to methodological constraints. The fact that most variables of our model were assessed using self-report measures makes it vulnerable to various biases, including response biases (e.g., leniency biases and acquiescence biases) and issues concerning the survey characteristics (e.g., the wording of items, answer formats, and the construct validity). For example, implicit and explicit coordination were found to be moderately correlated in the present study, sharing approximately 33% of their variance. This implies that the two coordination processes were not captured as opposing processes in the present study. The explicit coordination scale captured aspects such as the need for repeated explicit coordination within the team due to difficulties encountered. The correlation with implicit coordination implies that difficulties that arise can lead not only to an explicit exchange but also to an implicit exchange, i.e., the proactive help of others, for example. Furthermore, both scales were not validated in other studies. We thus see the potential for future research to use validated and more specific measures of explicit and implicit coordination.

One further problem that might go along with referent-shift consensus based self-reports (Chan, 1998) is the limited agreement among team members. Some of our measures showed relatively low ICC(2) values. Although this has made our analyses more conservative (Bliese, 1998; Hofmann and Jones, 2005), future research could therefore adopt a multi-level approach to team processes–as opposed to our aggregated approach (i.e., team mean values based on individual values)–and consider the perceptions of individual team members.

As an alternative to self-report measures, team processes should also be captured by focusing on time-based behavioral observation (Kolbe and Boos, 2019). A promising and unobtrusive way of capturing behavioral constructs at the individual and team level might be to collect observational data by videoing or by communication recording using wearable sensors (see Chaffin et al., 2015, for a review). However, since the use of wearable sensors in behavioral research has its own drawbacks and limitations (Chaffin et al., 2015), this method is more likely to complement, rather than replace, traditional methods (e.g., using a mixed methods approach; Molina-Azorin et al., 2017).

In addition, the use of student subjects may also limit the generalizability of our findings to other populations and non-academic settings. Although Wheeler et al. (2014) meta-analytically demonstrated that few differences between the observed correlations of student to non-student-recruited samples exist, student-recruited samples may lead to smaller effect sizes of observed statistical relationships, thus underestimating them.

Additionally, despite the considerable strengths of our study design and analysis, and our hypotheses are theoretically founded, the findings are correlational in nature, and our interpretations regarding causality are speculative. For example, future research should examine reverse causality in which the outcome (i.e., implicit coordination or performance) precedes and causes the exposure (reflection and explicit coordination). Longitudinal, multilevel study designs and experimental studies–although they have their own drawbacks–including quasi-experimental interrupted time series designs (Shadish et al., 2002) permit testing reverse causality hypotheses.

A final limitation of this study relates to the theory of change on which our model was based. The advantage of trajectories over traditional methods in longitudinal designs (see Selig and Little, 2012, for a critique of autoregressive and cross-lagged panel models) is that they allow to identify parameters of change processes (i.e., intercepts, slopes, and functional forms) and the relationship between them, providing a more sophisticated understanding of psychological concepts as they unfold over time. Although we examined an unstructured pattern of change, other non-linear patterns of change might also be possible. Future research should thus use intensive longitudinal designs (Walls and Schafer, 2006) to explore specific non-linear trajectories (e.g., quadratic and piecewise), which would require at least four measurement points. Also, the 2-months time frame was determined by the setting and design of the study and by the task, whereas the time lag was dependent on an event-contingent sampling protocol (Wheeler and Reis, 1991), based on discrete events (i.e., the two tasks). However, the event-contingent sampling protocol, which involves retrieval and reconstruction of experiences and behavior over a period of time and is thus susceptible to retrospective memory bias, does not make it possible to identify system transitions if the transitions are not synchronized with tasks. It would thus be illuminating to combine the event-contingent sampling protocol (i.e., meeting of teams) with a fixed or variable time-based protocol, which would enable future studies to provide a much more detailed picture of how experience and behavior are related in specific situations.

## Conclusion

In this study we adopted a three-wave longitudinal approach to understand the long-term reflection dynamic that occur within teams over a period of 2 months and how these changes are predictive of implicit and explicit coordination during later phases, and finally to improvements in team performance. Consistent with theory, teams working on heterogeneous tasks increased their reflection over time, and teams that had a lower baseline level of reflection showed a greater increase in reflection. More importantly, our results highlight the value of a high baseline level of reflection, as this was shown to foster implicit coordination in the later stages of teamwork, which in turn improved performance. Teams that started with a low baseline level of reflection and increased their reflection over time did not appear to be able to develop appropriate implicit coordination. Also, explicit coordination was shown to be negatively related to baseline levels of reflection and increases in team reflection and to be a factor that makes it more difficult for teams to improve team performance.

## Data Availability Statement

The raw data supporting the conclusions of this article will be made available by the authors, without undue reservation.

## Ethics Statement

Ethical review and approval was not required for the study on human participants in accordance with the local legislation and institutional requirements. The patients/participants provided their written informed consent to participate in this study.

## Author Contributions

MS contributed to conception and design of the study. UK performed the statistical analysis and wrote the first draft of the manuscript. MS, SK, and AF wrote sections of the manuscript. All authors contributed to manuscript revision, read, and approved the submitted version.

## Conflict of Interest

The authors declare that the research was conducted in the absence of any commercial or financial relationships that could be construed as a potential conflict of interest.

## Footnotes

1 We also acknowledged the potential role of team size (*M* = 3.33; *SD* = 0.49, range = 3–5) that may contribute to the level of coordination and/or performance. Since this inclusion does not alter our results, we omitted the control variable from our analyses.

2 A reviewer helpfully noted that not only the trajectory of reflection predicts coordination and changes in performance, but above and beyond the measure of reflection at Time 3. This model, that fitted the data well model [PPC 95% CI (−19.605, 27.941), PPP = 0.351, DIC = 1,500.146, RMSEA = 0.001, CFI = 1.000], revealed no significant relationships of reflection at T3 to performance improvement [β = −0.09, *PSD* = 0.16, CI (−0.41, 0.23), *ns*.], explicit coordination (β = 0.08, *PSD* = 0.29, CI [−0.38, 0.69], *ns*.), or implicit coordination [β = 0.08, *PSD* = 0.06, CI (−0.04, 0.23), *ns*.]. These results support the predictive validity of the reflection trajectories.

3 According to the recommendation of an reviewer, we have conducted a model in which explicit coordination predicted performance at T3, controlling for earlier performance at T1 and T2 (team task in the middle of the class; not relevant for our hypotheses). The results of the reasonable fitting model [PPC 95% CI (−5.220, 25.622), PPP = 0.086, DIC = 691.553, RMSEA = 0.101, CFI = 0.814] confirm our findings that explicit coordination is significantly negatively related to performance improvement.

## References

[B1] Ackerman P. L. (1987). Individual differences in skill learning: An integration of psychometric and information processing perspectives. *Psychol. Bull.* 102 3–27. 10.1037/0033-2909.102.1.3

[B2] Ancona D. G. Goodman P. S. Lawrence B. S. Tushman M. L. (2001). Time: a new research lens. *Acad. Manag. Rev.* 26 645–663. 10.5465/amr.2001.5393903

[B3] Bentler P. M. Hu P. (1995). *EQS: Structural equations program manual.* Los Angeles, CA: BMPD Statistical Software.

[B4] Bliese P. D. (1998). Group size, ICC values, and group-level correlations: A simulation. *Organ. Res. Methods* 1 355–373. 10.1177/109442819814001

[B5] Bliese P. D. (2016). *Multilevel modeling in R (2.6). A brief introduction to R, the multilevel package and the nlme package.* Available Online at: https://cran.r-project.org/doc/contrib/Bliese_Multilevel.pdf.

[B6] Bollen K. Curran P. (2006). *Latent curve models.* Hoboken, NJ: Wiley.

[B7] Brown R. D. Hauenstein N. M. A. (2005). Interrater agreement reconsidered: An alternative to the rwg indices. *Organ. Res. Methods* 8 165–184. 10.1177/1094428105275376

[B8] Bush J. T. LePine J. A. Newton D. W. (2018). Teams in transition: An integrative review and synthesis of research on team task transitions and propositions for future research. *Hum. Res. Manag. Rev.* 28 423–433. 10.1016/j.hrmr.2017.06.005

[B9] Butchibabu A. Sparano-Huiban C. Sonenberg L. Shah J. (2016). Implicit coordination strategies for effective team communication. *Hum. Factors* 58 595–610. 10.1177/0018720816639712 27113991

[B10] Carter S. M. West M. A. (1998). Reflexivity, effectiveness and mental health in BBC production teams. *Small Group Res.* 29 583–601. 10.1177/1046496498295003

[B11] Chaffin D. Heidl R. Hollenbeck J. R. Howe M. Yu A. Voorhees C. (2015). The promise and perils of wearable sensors in organizational research. *Organ. Res. Methods* 20 3–31. 10.1177/1094428115617004

[B12] Chan D. (1998). Functional relations among constructs in the same content domain at different levels of analysis: A typology of composition models. *J. Appl. Psychol.* 83 234–246. 10.1037/0021-9010.83.2.234

[B13] Chen J. Bamberger P. A. Song Y. Vashdi D. R. (2018). The effects of team reflexivity on psychological well-being in manufacturing teams. *J. Appl. Psychol.* 103 443–462. 10.1037/apl0000279 29239644

[B14] Cohen A. Doveh E. Nahum-Shani I. (2009). Testing agreement for multi-item scales with indices rwg(J) and ADM(J). *Organ. Res. Methods* 12 148–164. 10.1177/1094428107300365

[B15] Cronin M. A. Weingart L. R. Todorova G. (2011). Dynamics in groups: Are we there yet? *Acad. Manag. Ann.* 5 571–612. 10.1080/19416520.2011.590297

[B16] Curran P. J. Obeidat K. Losardo D. (2010). Twelve frequently asked questions about growth curve modeling. *J. Cogn. Dev.* 11 121–136. 10.1080/15248371003699969 21743795 PMC3131138

[B17] De Jong B. A. Elfring T. (2010). How does trust affect the performance of ongoing teams? The mediating role of reflexivity, monitoring, and effort. *Acad. Manag. J.* 53 535–549. 10.1016/j.outlook.2008.03.013 18501748

[B18] DeShon R. P. Kozlowski W. J. Schmidt A. M. Milner K. R. Wiechmann D. (2004). Multiple-goal, multilevel model of feedback effects on the regulation of individual and team performance. *J. Appl. Psychol.* 89 1035–1056. 10.1037/0021-9010.89.6.1035 15584840

[B19] Ellwart T. Konradt U. Rack O. (2014). Team mental models of expertise location: Validation of a field survey measure. *Small Group Res.* 45 119–153. 10.1177/1046496414521303

[B20] Gabelica C. Van den Bossche P. De Maeyer S. Segers M. Gijselaers W. (2014). The effect of team feedback and guided reflexivity on team performance change. *Learn. Instruct.* 34 86–96. 10.1016/j.learninstruc.2014.09.001

[B21] Gersick C. J. G. (1988). Time and transition in work teams: Toward a new model of group development. *Acad. Manag. J.* 31 9–41. 10.2307/256496

[B22] Grimm K. J. Ram N. (2012). “Growth curve modeling from a structural equation modeling perspective,” in *Handbook of Developmental Research Methods*, eds Laursen B. Little T. D. Card N. A. (New York, NY: The Guilford Press), 411–431.

[B23] Harrison D. A. Mohammed S. McGrath J. E. Florey A. T. Vanderstoep S. W. (2003). Time matters in team performance: Effects of member familiarity, entrainment, and task discontinuity on speed and quality. *Person. Psychol.* 56 633–669. 10.1111/j.1744-6570.2003.tb00753.x

[B24] Hofmann D. A. Jones L. M. (2005). Leadership, collective personality, and performance. *J. Appl. Psychol.* 90 509–522. 10.1037/0021-9010.90.3.509 15910146

[B25] Hollingshead A. B. (2001). Cognitive interdependence and convergent expectations in transactive memory. *J. Personal. Soc. Psychol.* 81 1080–1089. 10.1037/0022-3514.81.6.1080 11761309

[B26] Howard M. C. Hoffman M. E. (2018). Variable-centered, person-centered, and person-specific approaches: Where theory meets the method. *Organ. Res. Methods* 21 846–876. 10.1177/1094428117744021

[B27] Ilgen D. R. Hollenbeck J. R. Johnson M. Jundt D. (2005). Teams in organizations: From input-process-output models to IMOI models. *Annu. Rev. Psychol.* 56 517–543. 10.1146/annurev.psych.56.091103.070250 15709945

[B28] Kolbe M. Boos M. (2019). Laborious but elaborate: The benefits of really studying team dynamics. *Front. Psychol.* 10:1478. 10.3389/fpsyg.2019.01478 31316435 PMC6611000

[B29] Kolbe M. Burtscher M. Manser T. (2013). Co-ACT—A framework for observing coordination behavior in acute care teams. *BMJ Qual. Safety* 22 596–605. 10.1136/bmjqs-2012-001319 23513239

[B30] Konradt U. Eckardt G. (2016). Short-term and long-term relationships between reflection and performance in teams: Evidence from a four-wave longitudinal study. *Eur. J. Work Organ. Psychol.* 25 804–818. 10.1080/1359432X.2016.1160058

[B31] Konradt U. Otte K.-P. Schippers M. C. Steenfatt C. (2016). Reflexivity in teams: A review and new perspectives. *J. Psychol.* 150 153–172. 10.1080/00223980.2015.1050977 26457836

[B32] Konradt U. Schippers M. C. Garbers Y. Steenfatt C. (2015). Effects of guided reflexivity and team feedback on team performance improvement: the role of team regulatory processes and cognitive emergent states. *Eur. J. Work Organ. Psychol.* 24 777–795. 10.1080/1359432X.2015.1005608

[B33] Kozlowski S. W. (2015). Advancing research on team process dynamics: Theoretical, methodological, and measurement considerations. *Organ. Psychol. Rev.* 5 270–299. 10.1177/2041386614533586

[B34] LeBreton J. M. Senter J. L. (2008). Answers to 20 questions about interrater reliability and interrater agreement. *Organ. Res. Methods* 11 815–852. 10.1177/1094428106296642

[B35] LePine J. A. Piccolo R. F. Jackson C. L. Mathieu J. E. Saul J. R. (2008). A meta-analysis of teamwork processes: Tests of a multidimensional model and relationships with team effectiveness criteria. *Personnel Psychol.* 61 273–307. 10.1111/j.1744-6570.2008.00114.x

[B36] Lewis K. (2003). Measuring transactive memory systems in the field: Scale development and validation. *J. Appl. Psychol.* 88 587–604. 10.1037/0021-9010.88.4.587 12940401

[B37] Lewis K. (2004). Knowledge and performance in knowledge-worker teams: A longitudinal study of transactive memory systems. *Manag. Sci.* 50 1519–1533. 10.1287/mnsc.1040.0257 19642375

[B38] Lewis K. Lange D. Gillis L. (2005). Transactive memory systems, learning, and learning transfer. *Organ. Sci.* 16 581–598. 10.1287/orsc.1050.0143 19642375

[B39] Li C.-R. Li C.-X. Lin C.-J. (2020). Dynamics of the relationships between team reflexivity and team performance over a series of performance episodes. *Group Dynamics Theory Res. Practice* 10.1037/gdn0000144

[B40] Liang D. W. Moreland R. L. Argote L. (1995). Group versus individual training and group performance: The mediating role of transactive memory. *Personal. Soc. Psychol. Bull.* 21 384–393. 10.1177/0146167295214009

[B41] Lindell M. K. Brandt C. J. Whitney D. J. (1999). A revised index of agreement for multi-item ratings of a single target. *Appl. Psychol. Measur.* 23 127–135. 10.1177/01466219922031257

[B42] Lowry P. B. Roberts T. L. Romano N. C. Jr. (2013). What signal is your inspection team sending to each other? Using a shared collaborative interface to improve shared cognition and implicit coordination in error-detection teams. *Int. J. Hum. Comp. Stud.* 71 455–474. 10.1016/j.ijhcs.2012.11.004

[B43] Marks M. A. Mathieu J. E. Zaccaro S. J. (2001). A temporally based framework and taxonomy of team processes. *Acad. Manag. Rev.* 26 356–376. 10.5465/amr.2001.4845785

[B44] Marques-Quinteiro P. Curral L. Passos A. M. Lewis K. (2013). And now what do we do? The role of transactive memory systems and task coordination in action teams. *Group Dynamics Theory Res. Practice* 17 194–206. 10.1037/a0033304

[B45] Mathieu J. E. Heffner T. S. Goodwin G. F. Cannon-Bowers J. A. Salas E. (2005). Scaling the quality of teammates’ mental models: Equifinality and normative comparisons. *J. Organ. Behav.* 26 37–56. 10.1002/job.296

[B46] Mathieu J. E. Hollenbeck J. R. van Knippenberg D. Ilgen D. R. (2017). A century of work teams in the Journal of Applied Psychology. *J. Appl. Psychol.* 102 452–467. 10.1037/apl0000128 28150984

[B47] McGrath J. E. (1991). Time, interaction, and performance (TIP): A theory of groups. *Small Group Res.* 22 147–174. 10.1177/1046496491222001

[B48] Mohammed S. Dumville B. C. (2001). Team mental models in a team knowledge framework: Expanding theory and measurement across disciplinary boundaries. *J. Organ. Behav.* 22 89–106. 10.1002/job.86

[B49] Mohammed S. Ferzandi L. Hamilton K. (2010). Metaphor no more: A 15-year review of the team mental model construct. *J. Manag.* 36 876–910. 10.1177/0149206309356804

[B50] Molina-Azorin J. F. Bergh D. D. Corley K. G. Ketchen D. J. Jr. (2017). Mixed methods in the organizational sciences: Taking stock and moving forward. *Organ. Res. Methods* 20 179–192. 10.1177/1094428116687026

[B51] Moreland R. L. Myaskovsky L. (2000). Exploring the performance benefits of group training: Transactive memory or improved communication? *Organ. Behav. Hum. Decis. Process.* 82 117–133. 10.1006/obhd.2000.2891

[B52] Muthén B. Asparouhov T. (2012). Bayesian SEM: A more flexible representation of substantive theory. *Psychol. Methods* 17 313–335. 10.1037/a0026802 22962886

[B53] Muthén Muthén (1998–2020). *Mplus User’s Guide.* Los Angeles, CA: Muthén & Muthén.

[B54] Oertel R. Antoni C. H. (2015). Phase-specific relationships between team learning processes and transactive memory development. *Eur. J. Work Organ. Psychol.* 24 726–741. 10.1080/1359432X.2014.1000872

[B55] Otte K.-P. Knipfer K. Schippers M. C. (2019). “Team reflection: A catalyst of team development and the attainment of expertise,” in *The Oxford Handbook of Expertise: Research & Application*, eds Ward P. Schraagen J. M. Gore J. Roth E. (Oxford: Oxford University Press), 1–24.

[B56] Otte K.-P. Konradt U. Oldeweme M. (2018). Effective team reflection: the role of quality and quantity. *Small Group Res.* 49 739–766. 10.1177/1046496418804898

[B57] Podsakoff P. M. MacKenzie S. B. Lee J. Y. Podsakoff N. P. (2003). Common method Biases in behavioral research: A critical review of the literature and recommended remedies. *J. Appl. Psychol.* 88 879–903. 10.1037/0021-9010.88.5.879 14516251

[B58] Rico R. Sánchez-Manzanares M. Gil F. Gibson C. B. (2008). Team coordination processes: A team knowledge-based approach. *Acad. Manag. Rev.* 33 163–185. 10.5465/amr.2008.27751276

[B59] Riethmüller M. Castelao E. F. Eberhardt I. Timmermann A. Boos M. (2012). Adaptive coordination development in student anaesthesia teams: a longitudinal study. *Ergonomics* 55 55–68. 10.1080/00140139.2011.636455 22176484

[B60] Schippers M. C. (2014). Social loafing tendencies and team performance: The compensating effect of agreeableness and conscientiousness. *Acad. Manag. Learn. Educ.* 13 62–81. 10.5465/amle.2012.0191

[B61] Schippers M. C. Den Hartog D. N. Koopman P. L. (2007). Reflexivity in teams: A measure and correlates. *Appl. Psychol. Int. Rev.* 56 189–211. 10.1111/j.1464-0597.2006.00250.x

[B62] Schippers M. C. Den Hartog D. N. Koopman P. L. van Knippenberg D. (2008). The role of transformational leadership in enhancing team reflexivity. *Hum. Relat.* 61 1593–1616. 10.1177/0018726708096639

[B63] Schippers M. C. Edmondson A. C. West M. A. (2018). *Team reflexivity. In L. Argite & J. M. Levine, The Oxford Handbook of Group and Organizational Learning.* Oxford: Oxford University Press, 1–35.

[B64] Schippers M. C. Homan A. C. van Knippenberg D. (2013). To reflect or not to reflect: Prior team performance as a boundary condition of the effects of reflexivity on learning and final team performance. *J. Organ. Behav.* 34 6–23. 10.1002/job.1784

[B65] Schippers M. C. West M. A. Dawson J. F. (2015). Team reflexivity and innovation: The moderating role of team context. *J. Manag.* 41 769–788. 10.1177/0149206312441210

[B66] Schmutz J. B. Lei Z. Eppich W. J. Manser T. (2018). Reflection in the heat of the moment: The role of in-action team reflexivity in health care emergency teams. *J. Organ. Behav.* 39 749–765. 10.1002/job.2299

[B67] Selig J. P. Little T. D. (2012). “Autoregressive and cross-lagged panel analysis for longitudinal data,” in *Handbook of developmental research methods*, eds Laursen B. Little T. D. Card N. A. (New York City, NY: The Guilford Press), 265–278.

[B68] Shadish W. R. Cook T. D. Campbell D. T. (2002). *Experimental and quasi-experimental designs for generalized causal inference.* Houghton: Mifflin and Company.

[B69] Snijders T. A. B. Bosker R. J. (1999). *Multilevel analysis: An introduction to basic and advanced multilevel modeling.* London: Sage Publications.

[B70] Stewart G. L. (2006). A meta-analytic review of relationships between team design features and team performance. *J. Manag.* 32 29–55. 10.1177/0149206305277792

[B71] Swift T. A. West M. A. (1998). *Reflexivity and group processes: Research and practice.* Sheffield: The ESRC Centre for Organization and Innovation.

[B72] Tesler R. Mohammed S. Hamilton K. Mancuso V. McNeese M. (2018). Mirror, mirror: Guided storytelling and team reflexivity’s influence on team mental models. *Small Group Res.* 49 267–305. 10.1177/1046496417722025

[B73] Tjosvold D. (1991). *Team organization. An enduring competitive advantage.* New York: John Wiley and Sons.

[B74] Tjosvold D. Tang M. M. L. West M. (2004). Reflexivity for team innovation in China: The contribution of goal interdependence. *Group Organ. Manag.* 29 540–559. 10.1177/1059601103254911

[B75] van Ginkel W. Tindale R. S. van Knippenberg D. (2009). Team reflexivity, development of shared task representations, and the use of distributed information in group decision making. *Group Dynamics Theory Res. Practice* 13 265–280. 10.1037/a0016045

[B76] Villado A. J. Arthur W. Jr. (2013). The comparative effect of subjective and objective after-action reviews on team performance on a complex task. *J. Appl. Psychol.* 98 514–527. 10.1037/a0031510 23356248

[B77] Walls T. A. Schafer J. L. (2006). *Models for intensive longitudinal data.* Oxford: Oxford University Press.

[B78] Wegner D. M. (1986). “Transactive memory: A contemporary analysis of the group mind,” in *Theories of Group Behavior*, eds Mullen B. Goethals G. R. (Cham: Springer), 185–208.

[B79] West M. A. (2000). “Reflexivity, revolution, and innovation in work teams,” in *Product development teams*, Vol. 5 eds Beyerlein M. M. Johnson D. Beyerlein S. T. (Stamford: Connecticut: JAI Press), 1–29.

[B80] Wheeler A. R. Shanine K. K. Leon M. R. Whitman M. V. (2014). Student-recruited samples in organizational research: a review, analysis, and guidelines for future research. *J. Occup. Organ. Psychol.* 87 1–26. 10.1111/joop.12042

[B81] Wheeler L. Reis H. T. (1991). Self-recording of everyday life events: origins, types and uses. *J. Personal.* 59 339–354. 10.1111/j.1467-6494.1991.tb00252.x

[B82] Widmer P. S. Schippers M. C. West M. A. (2009). Recent developments in reflexivity research: A review. *Psychol. Everyday Activity* 2 2–11.

[B83] Wiedow A. Konradt U. Ellwart T. Steenfatt C. (2013). Direct and indirect effects of team process improvement on team outcomes: A multiple mediation analysis. *Group Dynamics Theory Res. Practice* 17 232–251. 10.1108/02683941111139029

[B84] Wittenbaum G. M. Vaughan S. I. Stasser G. (1998). “Coordination in task-performing groups,” in *Social psychological applications to social issues, Vol. 4. Theory and research on small groups*, eds Tindale R. S. Heath L. Edwards J. Posavac E. J. Bryant F. B. Suarez-Balcazar Y. (New York: Plenum Press), 177–204.

[B85] Woehr D. J. Loignon A. C. Schmidt P. B. Loughry M. L. Ohland M. W. (2015). Justifying aggregation with consensus-based constructs: a review and examination of cutoff values for common aggregation indices. *Organ. Res. Methods* 18 704–737. 10.1177/1094428115582090

[B86] Yang M. Schloemer H. Zhu Z. Lin Y. Chen W. Dong N. (2020). Why and when team reflexivity contributes to team performance: A moderated mediation model. *Front. Psychol.* 10:3044. 10.3389/fpsyg.2019.03044 32038407 PMC6985579

[B87] Zyphur M. J. Oswald F. L. (2015). Bayesian estimation and inference: A user’s guide. *J. Manag.* 41 390–420. 10.1177/0149206313501200

