# Supplementary material for: Teams in Transition: A Three-Wave Longitudinal Study of Reflection, Implicit and Explicit Coordination and Performance Improvements
Source: Front Psychol. 2021 Jun 7;12:677896. doi: 10.3389/fpsyg.2021.677896 (PMC8215207; doi:10.3389/fpsyg.2021.677896)
Supplement: Supplementary file 1 [file Data_Sheet_1.docx]

**Appendix**

*Implicit Coordination Scale Items Based on Rico et al. (2008)*

1. Team members pass on task-relevant information to others without being asked.
2. Team members provide others with feedback on their task performance before requesting it.
3. If someone in the team experiences high workload, others proactively complete his/her task.
4. Team members only support each other at work when being explicitly required (R).
5. Team members warn each other when they are likely to encounter problems on the team task.
6. As a team, we keep each other posted on the progress of the team activities.
7. If we have a deadline, we keep each other informed about the progress of the work.
8. If there are important changes in the team task, we make sure everyone is informed.
9. When I am under time pressure, other team members proactively help me.
10. As a team, we regularly synchronize our activities without the need of overt communication.
11. We proactively adapt to the needs of others, so that they can continue with their work.
12. When facing problem in the team task, I think how other team members will react.
13. I often consider other team members’ workload to see if they need some assistance.
14. When performing the team task, I anticipate how others will work.
15. I regularly think what I can do for my team that others are unlikely to do.
16. I anticipate what the contribution of everyone should be.
17. I anticipate on the work that needs to be done.

*Note.* All items use a 5-point disagree–agree response format, in which 1 = *totally disagree*, 2 = *disagree*, 3 = *neutral*, 4 = *agree*, and 5 = *totally agree*. (R) = reverse coded.
